# Mechano-Electric Coupling and Arrhythmogenic Current Generation in a Computational Model of Coupled Myocytes

**DOI:** 10.3389/fphys.2020.519951

**Published:** 2020-12-10

**Authors:** Viviane Timmermann, Andrew D. McCulloch

**Affiliations:** ^1^Simula Research Laboratory, Department of Computational Physiology, Fornebu, Norway; ^2^Departments of Bioengineering and Medicine, University of California San Diego, La Jolla, CA, United States

**Keywords:** mechno-electric feedback, intracellular calcium release, arrhythmia, calcium waves, computational model, rabbit, fibroblasts

## Abstract

A wide range of arrhythmogenic phenotypes have been associated with heterogeneous mechanical dyskinesis. Pro-arrhythmic effects are often associated with dysregulated intra-cellular calcium handling, especially *via* the development of intra- and inter-cellular calcium waves. Experimental evidence suggests that mechanical strain can contribute to the generation and maintenance of these calcium waves *via* a variety of mechano-electric coupling mechanisms. Most model studies of mechano-electric coupling mechanisms have been focused on mechano-sensitive ion channels, even though experimental studies have shown that intra- and inter-cellular calcium waves triggered by mechanical perturbations are likely to be more prevalent pro-arrhythmic mechanisms in the diseased heart. A one-dimensional strongly coupled computational model of electromechanics in rabbit ventricular cardiomyocytes showed that specific myocyte stretch sequences can modulate the susceptibility threshold for delayed after-depolarizations. In simulations of mechanically-triggered calcium waves in cardiomyocytes coupled to fibroblasts, susceptibility to calcium wave propagation was reduced as the current through the gap junction caused current drain from the myocytes. In 1D multi-cellular arrays coupled *via* gap junctions, mechanically-induced waves may contribute to synchronizing arrhythmogenic calcium waves and after-depolarizations.

## 1. Introduction

Altered myocardial mechanics in disease may contribute to the triggering or maintenance of life-threatening reentrant ventricular arrhythmias *via* a variety of mechano-electric coupling mechanisms. In particular, altered myocyte mechanics may cause pro-arrhythmic perturbations to cardiomyocyte electrophysiology (Franz et al., 1992; Kohl et al., 2011). These perturbations may generate currents that trigger arrhythmias or create a heterogenous myocardial electrical substrate that supports reentry (Taggart and Lab, 2008). While most model studies of mechano-electric coupling mechanisms have focused on mechano-sensitive ion channels (Peyronnet et al., 2016), experimental studies (ter Keurs et al., 2006b), and recent mathematical simulations (Timmermann et al., 2019) suggest that intra- and inter-cellular calcium waves triggered by mechanical perturbations are likely to be more prevalent pro-arrhythmic mechanisms in the diseased heart.

To understand the mechanisms of mechanically-triggered arrhythmogenic currents, we must consider both how electrical excitation activates calcium-dependent muscle contraction [excitation-contraction coupling (ECC)] and the reverse processes by which stretch alters membrane currents [mechano-electric coupling (MEC)] (Kohl et al., 1999). Under normal conditions, both pathways are important for the electrical regulation of cardiac performance underlying mechanical changes (Kohl et al., 1999). At the organ scale, electrical dyssynchrony alters mechanical contraction and may further exacerbate pump dysfunction in disease (Pfeiffer et al., 2014). At the (sub)cellular level, dysregulated calcium handling is associated with proarrhythmia, notably *via* the development of calcium waves (Kass and Tsien, 1982; Matsuda et al., 1982; Capogrossi and Lakatta, 1985; Capogrossi et al., 1986b; Backx et al., 1989). Thus, mechanically-triggered, calcium-mediated mechanisms are likely to contribute to arrhythmogenic phenotypes. Despite intensive investigations at different levels, from single ion channels (Kohl and Sachs, 2001) to tissue (ter Keurs et al., 1998, 2006a,b; Kohl et al., 2001), the contributions of MEC to arrhythmia generation is incompletely understood.

In addition to MEC mechanisms, fibrosis and electrical remodeling are crucial modulators of long term outcomes accompanying pathological mechanical changes in the heart. Up to 70% of the total cell number in healthy rodent myocardium is comprised of fibroblasts (MacCannell et al., 2007; Maleckar et al., 2009), which are thought to electrotonically interact with myocytes *via* gap-junctions (MacCannell et al., 2007; Maleckar et al., 2009). These functional, dynamic fibroblasts-to-myocyte interactions can modulate electrical excitability and action potential (AP) waveform (MacCannell et al., 2007; Maleckar et al., 2009). Thus, fibroblast-mediated electrical remodeling together with mechanical heterogeneities might exacerbate the susceptibility to pro-arrhythmic calcium wave propagation by providing arrhythmogenic substrate (MacCannell et al., 2007; Maleckar et al., 2009).

The aim of this study is to mechanistically explore the critical role of altered myocyte mechanics, mechano-electric coupling, calcium homeostasis, and myocyte coupling in the generation and maintenance of ventricular arrhythmias, to increase the understanding of the complex interaction and balance of calcium homeostasis and contractile function. To address this goal, we simulated a one-dimensional (1D) cardiac myocyte, by strongly coupling a zero-dimensional AP model to sarcomere mechanics. The coupled model was modified to represent the cardiac myocyte as a 1D string of bi-directional end-to-end interactions between sarcomeres. We introduced mechanical heterogeneities by allowing stochastic variations in sarcomere resting length and by subjecting the model to a wide range of stretch protocols. Coupling mechanically heterogeneous myocytes by gap junctions increased the order of dynamic instability to trigger potentially arrhythmogenic delayed after-depolarizations (DADs), while coupling fibroblasts to the myocyte had a stabilizing affect.

## 2. Materials and Methods

### 2.1. Electro-Mechanical Myocyte Models

#### 2.1.1. 0-Dimensional Myocyte Model

We developed a strongly coupled zero-dimensional (0D) computational model of rabbit cardiomyocyte electromechanics by coupling the well-established rabbit myocyte ionic model of Shannon et al. (2004) (distributed by Bers, 2002) with the myofilament mechanics model of Rice et al. (2008b) (downloaded from Rice et al., 2008a). The calcium transient generated by the ionic model, which represents normal calcium-induced calcium release (CICR) from the sarcoplasmic reticulum (SR) at the dyadic cleft, was input to the rabbit cardiac muscle contraction model. The formulation for cytosolic calcium binding to troponin C (TnC) in the mechanics model was used as input for the mechano-electric coupling of the mechanics model to the electrophysiology (EP) model. The contractile kinetics model described thin filament activation by intra-cellular calcium binding to TnC and the resulting crossbridge (XB) cycling.

In addition to strong electromechanical coupling, we incorporated strain-dependent changes in calcium affinity to the myofilaments to empirically approximate the triggers of mechanically-induced calcium waves. Using a parameter-fitting algorithm (Bueno-Orovio et al., 2008), the on- and off-rates for calcium binding to TnC and the constants for the strain-dependence of the XB cycling were reformulated to incorporate an exponential dependence on strain-rate. (A detailed description of the 0D model can be found in Timmermann et al., 2019).

#### 2.1.2. 1-Dimensional Myocyte Model

We extended the 0D electromechanical model of section 2.1.1 by arraying the 0D model into 50 uniform mechanically and resistively coupled units. Each unit represents a single sarcomere of the mechanical model and a single calcium-release unit (CRU) of the EP model. This linear array model was previously constructed to investigate the ability of sub-cellular mechanical perturbations to trigger and modulate calcium waves *via* strain-dependent thin filament calcium affinity (Timmermann et al., 2019). As reported by Timmermann et al. (2019), time-dependent calcium diffusion was implemented for the sub-sarcolemma (SS), SR, and cytosol. The diffusion time constants for intra-CRU diffusion in the longitudinal direction were adapted to reproduce a calcium wave velocity of ~100 $\frac{\mu \text{m}}{\text{s}}$ at Ca_o_ = 4 mM while increasing the magnitude of the calcium transient from the originally published value by Shannon et al. (2004). In particular, the calcium diffusion between the compartments of adjacent CRUs was incorporated when calculating the calcium concentrations as follows:

(1) $$\begin{array}{l} \begin{array}{l} \frac{{\text{dCa}}_{k}^{i}}{\text{dt}}=\frac{\hat{{\text{dCa}}_{k}^{i}}}{\text{dt}}+\frac{\left({\text{Ca}}_{k}^{i+1}+{\text{Ca}}_{k}^{i-1}-2\cdot {\text{Ca}}_{k}^{i}\right)}{\tau_{k}}\\ \;\forall i\in \left\{2,...,49\right\}\text{ and }k\in \left\{\text{SS},\text{SR},\text{cyto}\right\} \end{array} \end{array}$$

and for the boundaries:

(2) $$\begin{array}{l} \begin{array}{l} \frac{{\text{dCa}}_{k}^{1,50}}{\text{dt}}=\frac{\hat{{\text{dCa}}_{k}^{1,50}}}{\text{dt}}+\frac{\left({\text{Ca}}_{k}^{2,49}-{\text{Ca}}_{k}^{1,50}\right)}{\tau_{k}}\\ \;k\in \left\{\text{SS},\text{SR},\text{cyto}\right\} \end{array} \end{array}$$

with τ_SS_ = 0.4533 ms, τ_SR_ = 150 ms, τ_cyto_ = 1.2 ms, and $\frac{\hat{{\text{dCa}}_{k}^{1,50}}}{\text{dt}}$, *k* ∈ {SS, SR, cyto}, the time-dependent changes calcium concentrations as in Shannon et al. (2004). (A detailed description of the one dimensional (1D) model can be found in Timmermann et al., 2019).

For this study, the model was extended by calculating the membrane potential in each of the 50 CRUs to better represent the electrical conductance of a myocyte. The first CRU in the myocyte was stimulated with a 1,000 mV pulse for 3 ms, triggering a electrical stimulation of the entire cell. Using a parameter-fitting algorithm (Bueno-Orovio et al., 2008), the time constant for voltage transfer between adjacent CRUs and the electrical pulses was chosen to allow electrical propagation while retaining a similar magnitude of the AP upstroke as reported in the original ionic model (Shannon et al., 2004).

(3) $$\begin{array}{l} \begin{array}{l} \frac{{\text{dV}}_{\text{m}}^{1}}{\text{dt}}=-\frac{1}{{\text{C}}_{\text{m}}}\left({\text{I}}_{\text{m}}^{i}+{\text{I}}_{\text{stim}}\right)+\frac{\left({\text{V}}_{\text{m}}^{2}-{\text{V}}_{\text{m}}^{1}\right)}{\tau_{{\text{V}}_{\text{m}}}}\\ \frac{{\text{dV}}_{\text{m}}^{i}}{\text{dt}}=-\frac{1}{{\text{C}}_{\text{m}}}\left({\text{I}}_{\text{m}}^{i}\right)+\frac{\left({\text{V}}_{\text{m}}^{i+1}+{\text{V}}_{\text{m}}^{i-1}-2\cdot {\text{V}}_{\text{m}}^{i}\right)}{\tau_{{\text{V}}_{\text{m}}}}\\ \frac{{\text{dV}}_{\text{m}}^{50}}{\text{dt}}=-\frac{1}{{\text{C}}_{\text{m}}}\left({\text{I}}_{\text{m}}^{i}\right)+\frac{\left({\text{V}}_{\text{m}}^{\text{49}}-{\text{V}}_{\text{m}}^{50}\right)}{\tau_{{\text{V}}_{\text{m}}}}\\ \;\forall i\in \left\{2,...,49\right\} \end{array} \end{array}$$

with C_m_ membrane capacitance, $\tau_{{\text{V}}_{\text{m}}}=1e^{-5}$ ms conductance propagation coefficient, I_stim_ the stimulus current, and I^*i*^ the total membrane current in CRU *i* ∈ {1, ..., 50}.

Furthermore, transmembrane currents with variable conductances were adapted to represent the mean experimentally reported values at the three cycle lengths 400, 600, and 1,000 ms as reported in Gemmell et al. (2016). In particular, the conductance of the transient outward potassium current (I_*to*_) was increased by 30%, the conductance of slow delayed rectifier current (I_*Ks*_) was decreased by 15%, the inward rectifier current (I_*K*1_) was decreased by 30%, and the sodium-potassium pump current was increased by 15%.

In addition to interactions between adjacent ionic CRUs in this spatially explicit model, a single sarcomere was coupled to each EP model. We used the strong coupling approach of Timmermann et al. (2019) to represent mechanical interaction between neighboring sarcomeres. Therefore, fractional changes in length between adjacent sarcomeres were included:

(4) $$\begin{array}{l} \begin{array}{l} \frac{{\text{dSL}}_{j}^{i}}{\text{dt}}=2\cdot \hat{{\text{dSL}}_{j}^{i}}-\left(\hat{{\text{dSL}}_{j}^{i+1}}+\hat{{\text{dSL}}_{j}^{i-1}}\right)\\ \;\forall i\in \left\{2,3,...,49\right\}\;\forall j\in \left\{1,2,3\right\} \end{array} \end{array}$$

with $\hat{{\text{dSL}}_{j}^{i}}$ the isotonic length change of sarcomere *i* of myocyte *j*.

The sarcomeres at the boundaries of each myocyte, interacted only with one other sarcomere of the same myocyte. To incorporate mechanical interactions between adjacent myocytes, adjacent sarcomeres of neighboring myocytes affected each other by their scaled fractional change in length

(5) $$\begin{array}{l} \begin{array}{l} \frac{{\text{dSL}}_{1}^{1}}{\text{dt}}=\hat{{\text{dSL}}_{1}^{1}}-\hat{{\text{dSL}}_{1}^{\text{2}}}\\ \frac{{\text{dSL}}_{j}^{1}}{\text{dt}}=\hat{{\text{dSL}}_{j}^{1}}-\left(\hat{{\text{dSL}}_{j}^{\text{2}}}+\hat{{\text{dSL}}_{j-1}^{50}}\right)\\ \;\forall j\in \left\{2,...,n\right\}\\ \frac{{\text{dSL}}_{j}^{50}}{\text{dt}}=\hat{{\text{dSL}}_{j}^{50}}-\left(\hat{{\text{dSL}}_{j}^{\text{49}}}+\hat{{\text{dSL}}_{j+1}^{1}}\right)\\ \;\forall j\in \left\{1,...,n-1\right\}\\ \frac{{\text{dSL}}_{n}^{50}}{\text{dt}}=\hat{{\text{dSL}}_{n}^{50}}-\hat{{\text{dSL}}_{n}^{\text{49}}} \end{array} \end{array}$$

with $\hat{{\text{dSL}}_{j}^{1,50}}$ the isotonic length change of the first (1) or the last (50) sarcomere, respectively, of the *j*th myocyte, and *n* = 3 or 6 for the number of coupled myocytes.

#### 2.1.3. 1-Dimensional Multi-Cellular Model Coupled *via* Gap Junctions

To adapt the 1D electromechanical myocyte model for simulations of multi-cellular interactions, 3 myocytes were coupled *via* gap junctions. Each of the three myocytes in the coupled simulation was modeled as 1 of the 10 different single myocyte models described in section 2.1.2. Ten separate multi-cellular simulations with varying combinations of 3 of these 10 different single myocyte conditions were analyzed. The modeled combinations are given in Supplementary Tables 6, 7. To reproduce a conductance velocity of approximately 0.5 $\frac{\mu \text{m}}{\text{s}}$, the value of gap junction conductance was g_gap_ = 400 nS. The corresponding equations for the linear gap junction current from the *j*th to the (*j* + 1)th cell were as follows:

(6) $$\begin{array}{l} \begin{array}{l} \;{\text{I}}_{\text{gap}}^{j}={\text{g}}_{\text{gap}}\left(\hat{{\text{V}}_{\text{m},j}^{50}}-\hat{{\text{V}}_{\text{m},j+1}^{1}}\right)\\ \;\frac{{\text{dV}}_{\text{m},j}^{50}}{\text{dt}}=\frac{\hat{{\text{dV}}_{\text{m},j}^{50}}}{\text{dt}}+{\text{I}}_{\text{gap}}^{j}\\ \frac{{\text{dV}}_{\text{m},j+1}^{50}}{\text{dt}}=\frac{\hat{{\text{dV}}_{\text{m},j+1}^{50}}}{\text{dt}}-{\text{I}}_{\text{gap}}^{j}\\ \;\forall j\in \left\{1,...,n-1\right\} \end{array} \end{array}$$

with $\frac{\hat{{\text{V}}_{\text{m},j}^{50}}}{dt}$, $\frac{\hat{{\text{V}}_{\text{m},j}^{1}}}{\text{dt}}$, the change in membrane potential of the last CRU of myocyte *j* and the first CRU of myocyte (*j* + 1) in absence of the gap-junctional coupling, *n*, the number of myocytes, and $\frac{{\text{V}}_{\text{m},j}^{50}}{\text{dt}}$, $\frac{{\text{V}}_{\text{m},j}^{1}}{\text{dt}}$, the change in the potential of the membrane at last sarcomere of myocyte *j* and at the first sarcomere of myocyte (*j* + 1) in the presence of gap-junctional coupling.

For these multi-cellular simulations, the first CRU of the first myocyte (left boundary of the string of myocytes) was electrically stimulated with a 1,000 mV pulse for 3 ms. The adjacent myocytes were stimulated by the coupling *via* gap junctions.

### 2.2. Incorporation of Inter-cellular Mechanical Heterogeneities

We introduce mechanical heterogeneities by allowing sarcomeres within a single myocyte to have different resting lengths. To induce sarcomere heterogeneity, we deviated from the average resting length of 1.89 μm (Rice et al., 2008b), by 10% which are in the physiological range measured in myofibrils by Rassier et al. (2003). Therefore, for 10 random variations, the resting lengths of the 50 sarcomeres were chosen from the interval [1.701, 2.079 μm] (as shown in Supplementary Table 1) using a uniform random number generator (the *rand* function of MATLAB).

### 2.3. Incorporation of Fibroblasts

To simulate myocyte-fibroblast interactions, the active version 1 model of Maleckar et al. (2009) was incorporated to the electromechanics model. The current conductance of the fibroblasts is described by five time- and voltage-dependent equations in addition to the gap junction which allows for Na^+^ and K^+^-ions to move between the myocyte and the fibroblast (for a detailed description see Maleckar et al., 2009). Originating from the fibroblast model of MacCannell et al. (2007), the active model of Maleckar et al. (2009) includes the fibroblast membrane capacitance and four fibroblast ionic currents: time- and voltage-dependent fibroblast K^+^ current (I_KV_), inward-rectifying ${\text{K}}_{1}^{+}$ current (I_K1_) which is expressed in cardiac myofibroblasts, Na^+^-K^+^ pump current (I_NaK_) which has expressed K^+^ channels resulting in K^+^ fluxes, and background Na^+^ current (I_b,Na_) to balance the Na^+^ efflux from the Na^+^-K^+^ pump activity (for a detailed description see MacCannell et al., 2007).

Each myocyte was coupled to 1, 2, or 3 fibroblasts. Each fibroblast was randomly assigned to one CRU at 1 of the last 5 CRUs at one of the ends of the myocyte due to its relatively small size compared with the myocyte since fibroblast locate close to the gap junctions between myocytes (Goldsmith et al., 2004; Ongstad and Kohl, 2016). For each coupled fibroblast the value of the linear, fixed resistance (G_f,gap_) varied randomly in the interval between the low and high ends of the range of G_f,gap_, 0.5 and 8.0 nS as reported in Maleckar et al. (2009). Each of the 10 myocytes described in section 2.2, we assigned a random variation of the fibroblast locations (as shown in Supplementary Table 2).

### 2.4. Pacing and Stretch Protocols

In the electromechanics model we mimic various heterogeneities by subjecting the model to a wide range of stretch protocols. Myocyte stretch was modeled as an increase in sarcomere length. All stretch protocols of single cell and multi-cellular simulations were run for 200 beats to reach limit cycle. The limit cycle was ascertained by comparing the upstroke, plateau, and repolarization phase of the last two APs by calculating the mean squared error over time between both curves until mean squared error <0.01.

Under calcium overload conditions, calcium sparks may be more likely to trigger propagated waves (Backx et al., 1989; Cheng et al., 1996). Therefore, to study calcium sparks and propagating calcium waves, all simulations were performed by increasing extracellular calcium concentration in 0.1 mM steps from Ca_o_ = 2.0 mM to Ca_o_ = 4.5 mM, which results in increased calcium transient in all our simulations from the reported values of Shannon et al. (2004). Spontaneous calcium waves with wave velocities of ~ 100 $\frac{\mu \text{m}}{\text{s}}$ have been reported at low extracellular calcium concentrations as 1.8–2.0 mM (Takamatsu and Wier, 1990; Wier and Blatter, 1991) while others reported wave velocities of ~72 $\frac{\mu \text{m}}{\text{s}}$ at Ca_o_ = 2 mM, ~80 $\frac{\mu \text{m}}{\text{s}}$ at Ca_o_ = 5 mM, and ~90 $\frac{\mu \text{m}}{\text{s}}$ at Ca_o_ = 15 mM (Capogrossi et al., 1986a; Cheng et al., 1996).

**Figure 1:**
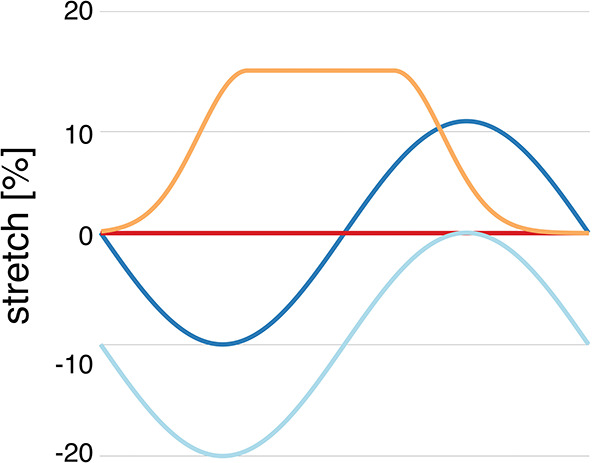
Myocyte stretch protocols. Stretch pattern H (light blue) approximates normal isotonic myocyte shortening with a sinusoidal function. Protocol I (red) is sarcomere isometric contraction. Protocol S (dark blue) simulates late systolic stretch by shifting the sine wave upwards. Protocol P (orange) simulates systolic lengthening such as occurs in acutely ischemic regions.

We refer to normal healthy contraction as protocol H, a sinusoidal function decreasing to 20% compression, and isometric contraction as protocol I. Pathophysiological stretches were represented by a sinusoidal function decreasing to 10% compression in diastole and increasing to 10% stretch in systole (protocol S) and an exponentially increasing function, increasing from 0 to 15% at the start of contraction, and then kept constant until it decreased exponentially at the time of relaxation (protocol P) (as shown in Figure 1). These protocols were used both for simulations of single and coupled myocytes both with and without coupled fibroblasts. Additionally, all four stretch protocols were applied to the 10 different single myocyte models generated in section 2.2 as well as the 10 different single myocyte models with 1, 2, or 3 coupled fibroblasts as described in section 2.3.

All single cell simulations were run in MATLAB R2018a using *ode15s* to solve the ordinary differential equation system, while multi-myocyte simulations were run in C++ using *odeint*.

### 2.5. Data Analysis

The statistical tests are presented as mean ± standard deviation calculated in MATLAB R2018a using the functions *mean* and *std*. The data was analyzed in MATLAB R2018a with the functions *anova1* and *multicompare*, a one-way analysis of variance with pairwise comparison of the group means preconditioned by the Bonferroni method. A data point was assumed to be an outlier if it did not lie within three standard deviations of the mean (three-sigma rule). Significant differences between were illustrated by asterisks with three asterisks indicating a *p* < 0.01, two a *p* < 0.05, and one a *p* < 0.1. Statistical tests were assessed for all simulations, even though some significant differences are far below the accuracy of experimental measurement techniques. To measure the inter-cellular dyssynchrony of intra-cellular calcium dynamics, we evaluated the standard deviation between cells of the mean time-to-peak of the intra-cellular calcium transient within each cell.

### 2.6. Data Availability

The datasets for this study can be found in the repository: https://github.com/cmrglab/1D_MEC.git.

## 3. Results

### 3.1. The Occurrence of Calcium Waves and Wave Velocity Is Dependent on the Stretch Pattern and Ca_o_

For all single myocyte simulations, we examined susceptibility to the generation of calcium waves after both pacing and stretching were stopped.

Even though some studies (Takamatsu and Wier, 1990; Wier and Blatter, 1991) have reported propagating calcium waves at low calcium concentrations as Ca_o_ = 1.8–2.0 mM, we did not observe spontaneous calcium waves in our model at Ca_o_ = 2.0 mM. Therefore, we increased the extracellular calcium concentration in 0.1 mM steps until in 3 of 10 control experiments (no stretch, stretch pattern I) spontaneously propagating calcium waves were observed at Ca_o_ = 4.1 mM. These findings are largely consistent with many experimental reports of calcium waves, in which the extracellular calcium concentrations varied between ~2.0 mM (Takamatsu and Wier, 1990; Wier and Blatter, 1991) and 15 mM (Capogrossi et al., 1986a; Cheng et al., 1996).

**Figure 2:**
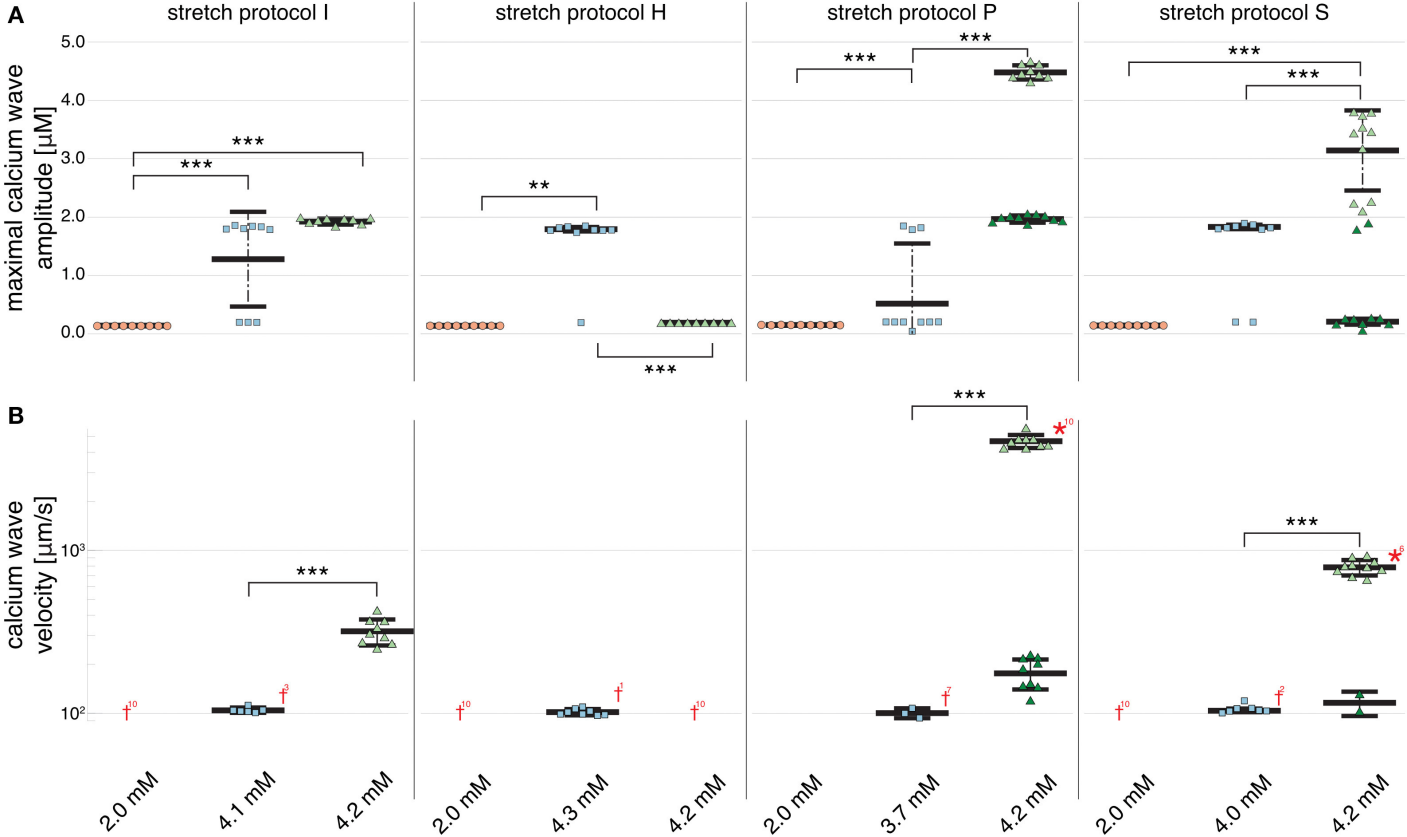
Maximal calcium wave amplitude magnitude and calcium wave velocity in cardiomyocyte simulations at different extracellular calcium concentrations for control (no stretch protocol I), and stretch patterns H, S, and P. **(A)** The magnitude of the measured maximal calcium wave amplitude increased significantly for all stretch patterns from Ca_o_ = 2.0 mM (red data points) to the extracellular calcium concentration at which a calcium wave occurred (blue data points), and 4.2 mM (green data points). Red daggers indicate that no calcium waves occurred in the data set, while red asterisks indicate that the calcium waves triggered additional beats. The related numbers specifies the amount of occurrences within the data set. The data points in light colors specify the first spontaneous event and dark colors are used for the following spontaneous event. **(B)** The calcium wave velocity increased for all simulations from Ca_o_ = 2.0 mM (no waves) to 4.2 mM and from control, to stretch patterns H, to S, and to P. **, *** indicate the statistical significance with *p* < 0.05 and *p* < 0.1, respectively.

Figure 2B shows that no calcium waves were observed for the control experiments at Ca_o_ = 2.0 mM (indicated by red daggers). For isotonic stretch simulations, the first calcium waves occurred at Ca_o_ = 4.3 mM, while all for all other stretch patterns lower calcium concentrations were sufficient to trigger spontaneously propagating calcium waves (see Figure 2B). At Ca_o_ = 4.2 mM, stretch pattern P generated the fastest calcium waves which always triggered an additional beat (indicated by the red star in Figure 2B) and propagated with a mean velocity of 4596.1 ± 408.22 $\frac{\mu \text{m}}{\text{s}}$. This additional beat was followed by a slow calcium wave which propagated with a 175.58 ± 37.98 $\frac{\mu \text{m}}{\text{s}}$ (shown in Supplementary Table 3). Even though the extracellular calcium concentration at which a calcium wave occurred for the different stretch patterns varied between Ca_o_ = 3.7 and 4.3 mM, the wave velocity was ~100 $\frac{\mu \text{m}}{\text{s}}$ for all stretch patterns. While the velocity of the first calcium wave was similar between stretch patterns, the maximal calcium wave amplitude (shown in Figure 2A) varied depending on the stretch pattern due to the extracellular calcium concentration. The lowest mean of the maximal calcium wave amplitude was observed for stretch pattern H with a magnitude of 0.15 ± 0.0 μM and the highest maximal calcium wave amplitude for stretch pattern P with a magnitude of 4.46 ± 0.13 μM.

**Figure 3:**
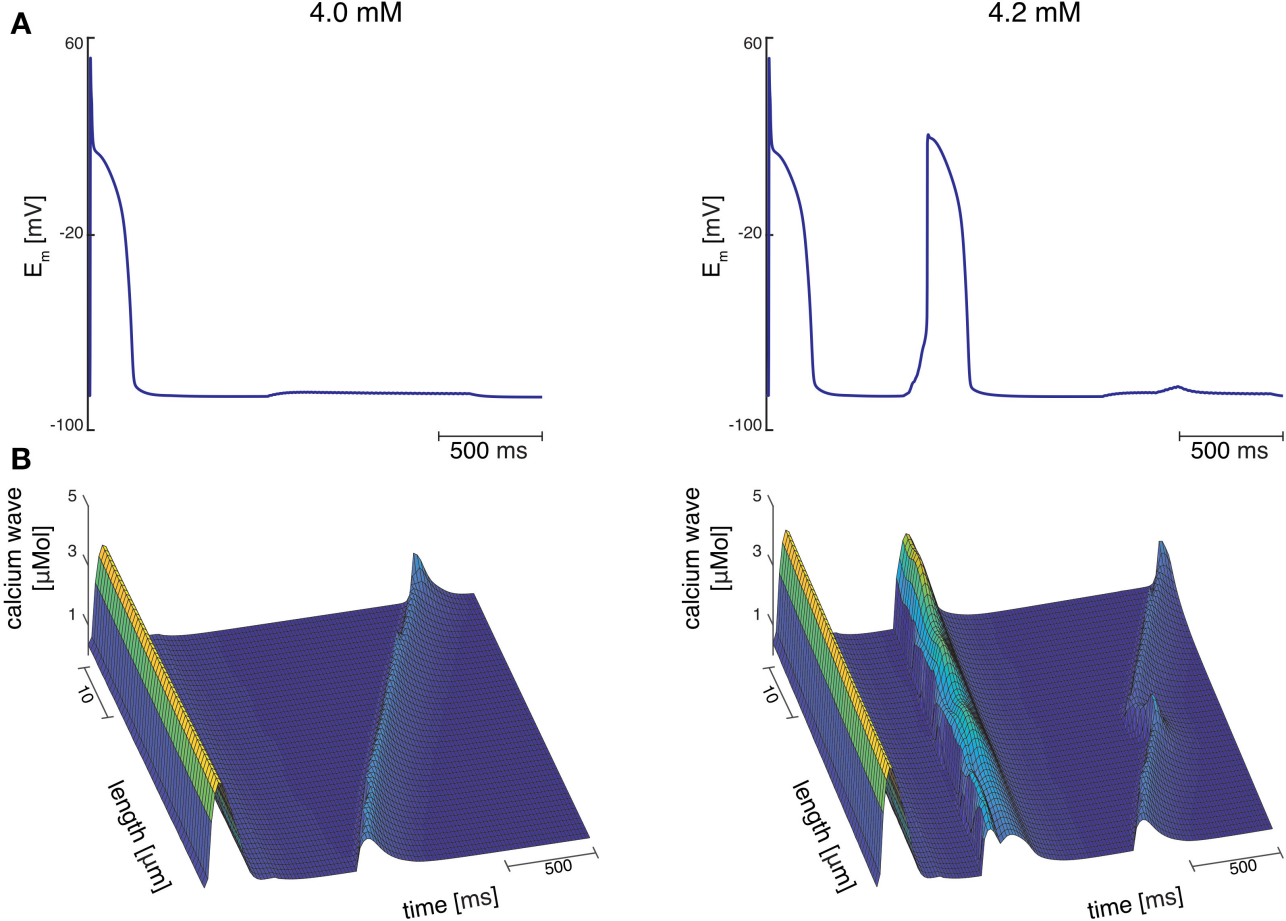
**(A)** Membrane potential for stretch pattern S of myocyte 3 at Ca_o_ = 4.0 mM and Ca_o_ = 4.2 mM (from left to right). After one stimulated beat, at Ca_o_ = 4.0 mM a calcium-mediated suprathreshold DAD occurred and at Ca_o_ = 4.2 mM a calcium-mediated additional beat occurred followed by a calcium wave. **(B)** Associating calcium wave for the supratheshold DAD, and the additional beat followed by a calcium wave (from left to right) at Ca_o_ = 4.0 mM and Ca_o_ = 4.2 mM, respectively.

At Ca_o_ = 4.2 mM, the greatest variation in calcium wave velocity was observed for stretch pattern P with a velocity of 4005.5 ± 408.8 $\frac{\mu \text{m}}{\text{s}}$. For all simulations at Ca_o_ = 4.2 mM, the resulting calcium waves induced suprathreshold DADs. For stretch pattern P in all 10 myocytes, a fast calcium wave triggered an extra beat followed by a slow propagating calcium wave, while for stretch pattern S in 6 of 10 myocytes, an additional beat and slow wave was observed in only two simulations. An example for the different calcium-mediated effects on AP are shown in Figure 3A and the associated calcium waves responsible for the changes in AP are illustrated in Figure 3B. At Ca_o_ = 4.2 mM a fast propagating wave triggered an additional beat, which was followed by a slower propagating wave. As described in Timmermann et al. (2019), calcium waves arose from spontaneous calcium release from the SR as a result of SR calcium overload. SR calcium overload resulted from stretch-dependent myofilament calcium dissociation that was sufficient to alter cytosolic calcium dynamics.

Figure 2B shows that an increase in calcium wave velocity is associated with an increase in maximal calcium wave amplitude. For control (stretch protocol I), the calcium wave amplitude significantly (*p* < 0.01) increased for experiments at Ca_o_ = 4.1 mM to Ca_o_ = 4.2 mM from 1246.0 ± 819.0 $\frac{\mu \text{m}}{\text{s}}$ to 1896.3 ± 52.0 $\frac{\mu \text{m}}{\text{s}}$ (see Figure 2A). Similarly, the maximal calcium wave amplitude for simulations for stretch patterns S and P increased significantly (*p* < 0.01) for experiments at which calcium waves first occurred to Ca_o_ = 4.2 mM by 637.25, 217.33%, respectively. (Mean and standard deviation of the maximal calcium wave amplitude magnitude and calcium wave velocity can be found in Supplementary Table 3).

### 3.2. Fibroblasts Function as Current Drains in Simulations of Spontaneous Calcium Waves

**Figure 4:**
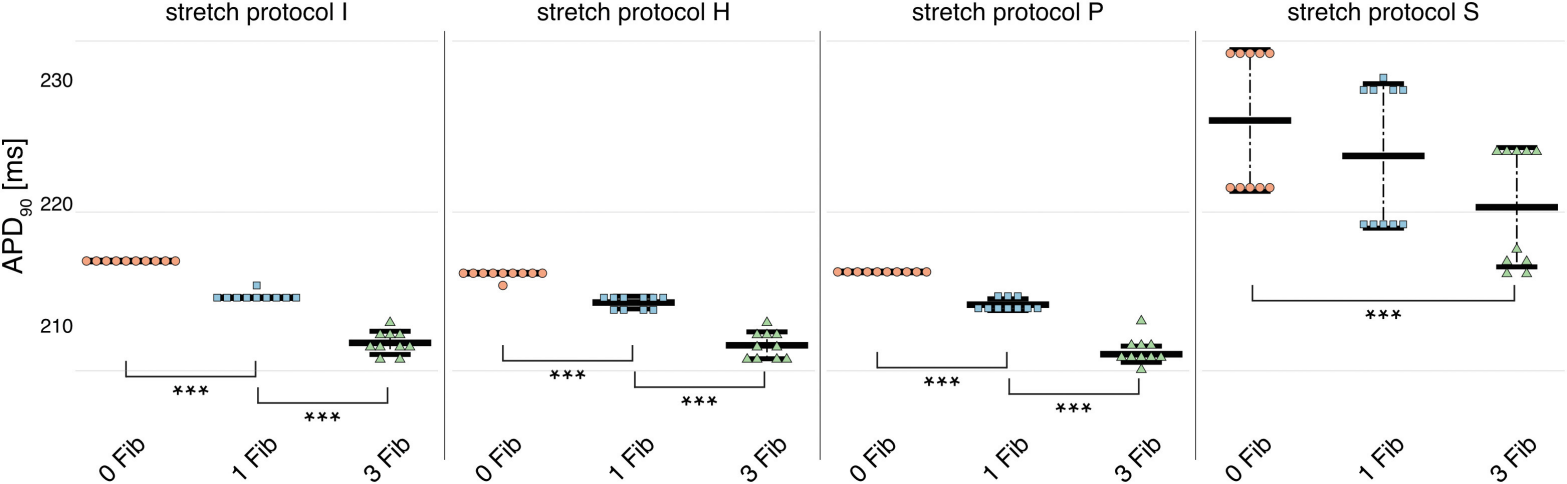
APD_90_ for simulations of myocytes with no (red data points), 1 (blue data points), and 3 coupled fibroblast (green data points) compared with control (no coupled fibroblast) for no contraction and stretch patterns H, S, and P. APD_90_ was significantly reduced for all simulations of myocytes with coupled myocytes for the different stretch patterns.

Owing to the large number of fibroblasts in the myocardium, we investigated their role in modulating electrophysiological instabilities. For all stretch patterns of myocytes with coupled fibroblasts, the current through the gap junction provided a drain of electric charge from the cardiomyocyte. Therefore, we were able to replicate a significant (*p* < 0.01) shortening of APD_90_ (see Figure 4 and in Supplementary Table 4) as reported by MacCannell et al. (2007).

**Figure 5:**
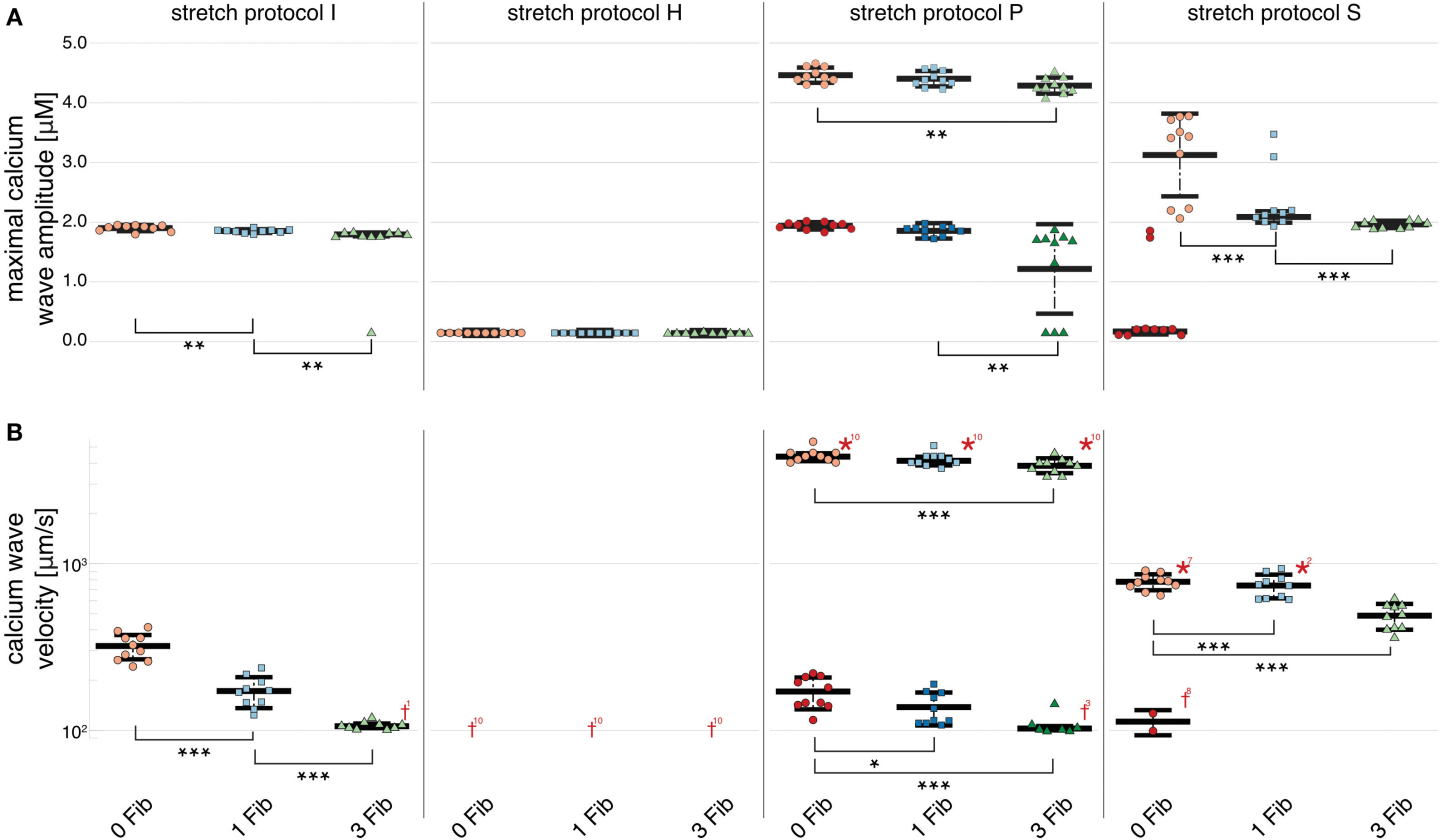
Maximal calcium wave amplitude and calcium wave velocity for simulations for control (red data points), and myocytes with one (blue data points) and two (green data points) coupled fibroblasts. The data points in light colors specify the first spontaneous event and dark colors are used for the following spontaneous event. **(A)** For stretch patterns I (control), S, and P the maximal calcium wave amplitude significantly (*p* < 0.01) decreased the more fibroblasts were coupled to the myocyte. **(B)** The wave velocity significantly (*p* < 0.01) decreased for stretch patterns I (control), S, and P for no fibroblast coupled to three coupled fibroblasts. *, **, *** indicate the statistical significance with *p* < 0.01, *p* < 0.05, and *p* < 0.1, respectively.

For simulations of calcium-mediated DADs the current through the gap junction between the fibroblast and the myocyte supplied an electric discharge from the cardiomyocyte as illustrated by the significantly (*p* < 0.01) decreasing maximal calcium wave amplitude in Figure 5A. Associated with decreased maximal calcium wave amplitude magnitude, the velocity of the induced calcium wave decreased significantly (*p* < 0.01) as more fibroblasts were coupled to the myocyte (see Figure 5B). For no stretch, in simulations with 3 coupled fibroblasts in 1 of 10 myocytes, no wave occurred, while for simulations of stretch patterns P and S, calcium waves were present. However, for simulations of stretch pattern P with three coupled fibroblasts no slow calcium waves occurred after the triggered beat. calcium waves in myocytes coupled to three fibroblasts did not elicit extra beats in simulations of stretch pattern S.

The calcium waves in our computational model are triggered through spontaneous calcium release from the SR, which in turn triggers an increase in membrane potential and potentially, a DAD. Therefore, the coupled fibroblasts not only drain electrical charge and reduce the APD_90_ during normal pacing, but also slow calcium waves as charge is drained from the associated calcium-mediated suprathreshold DAD.

### 3.3. Calcium Waves Are More Synchronized in Multi-Cellular Simulations

**Figure 6:**
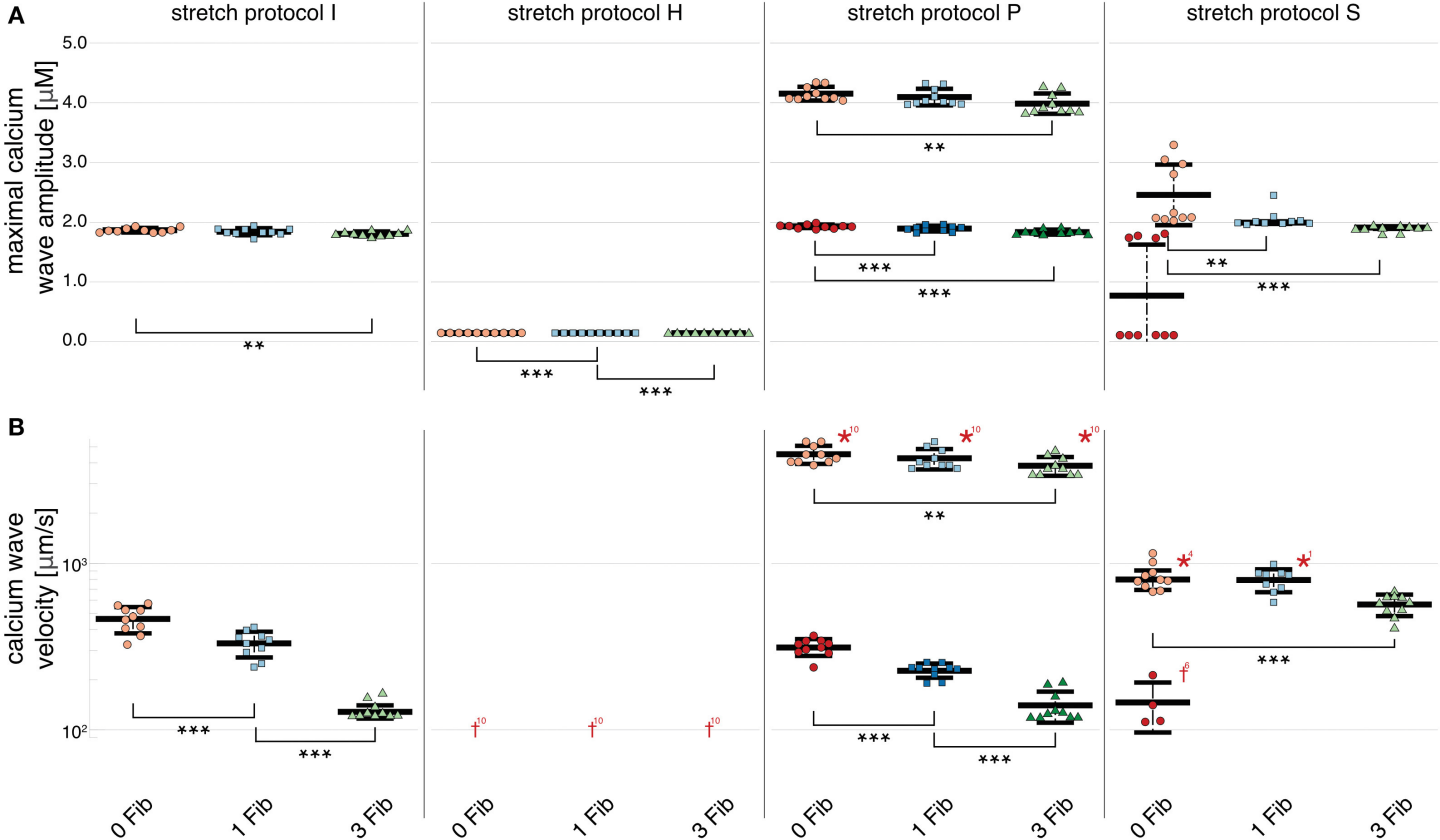
Maximal calcium wave amplitude and calcium wave velocity for simulations of three coupled cardiomyocytes. **(A)** For all stretch patterns but H (from left to right: control, H, S, and P) the maximal calcium wave amplitude significantly (*p* < 0.01) decreased the more fibroblasts were coupled to the multi-cellular string (control red data points, 1 coupled fibroblast blue data points, and 3 coupled fibroblasts green data points; the data points in light colors specify the first spontaneous event and dark colors are used for the following spontaneous event). **(B)** The wave velocity significantly (*p* < 0.01) decreased for all stretch patterns from no coupled fibroblast to three coupled fibroblasts. *, **, *** indicate the statistical significance with *p* < 0.01, *p* < 0.05, and *p* < 0.1, respectively.

In simulations of 3 and 6 cardiomyocytes coupled *via* gap junctions, calcium wave velocity and calcium wave amplitude of the multi-cellular simulation are given as the mean of the individually measured observed calcium wave velocities and calcium wave amplitudes of each single, coupled cell. Calcium wave velocity slightly decreased for all stretch patterns and for fibroblast coupling compared to single myocytes (see Figures 5, 6, respectively). The results shown here were computed for three coupled myocytes but no differences were observed when we repeated these calculations with six coupled cells (shown in Supplementary Figure 1). Coupled myocytes behaved similarly to single myocytes. Maximal calcium wave amplitude varied between simulations of no stretch, and stretch patterns P and H with the highest maximal calcium wave amplitude occurring for stretch pattern P, which coincides with the fastest observed wave velocity (as shown in Figure 6). With stretch pattern S, fewer additional beats were triggered in the coupled myocyte simulations, showing how multi-cellular tissue can be less susceptible to extra beats than isolated cells due to the slowing and synchronization of calcium waves by electrotonic coupling effects.

**Figure 7:**
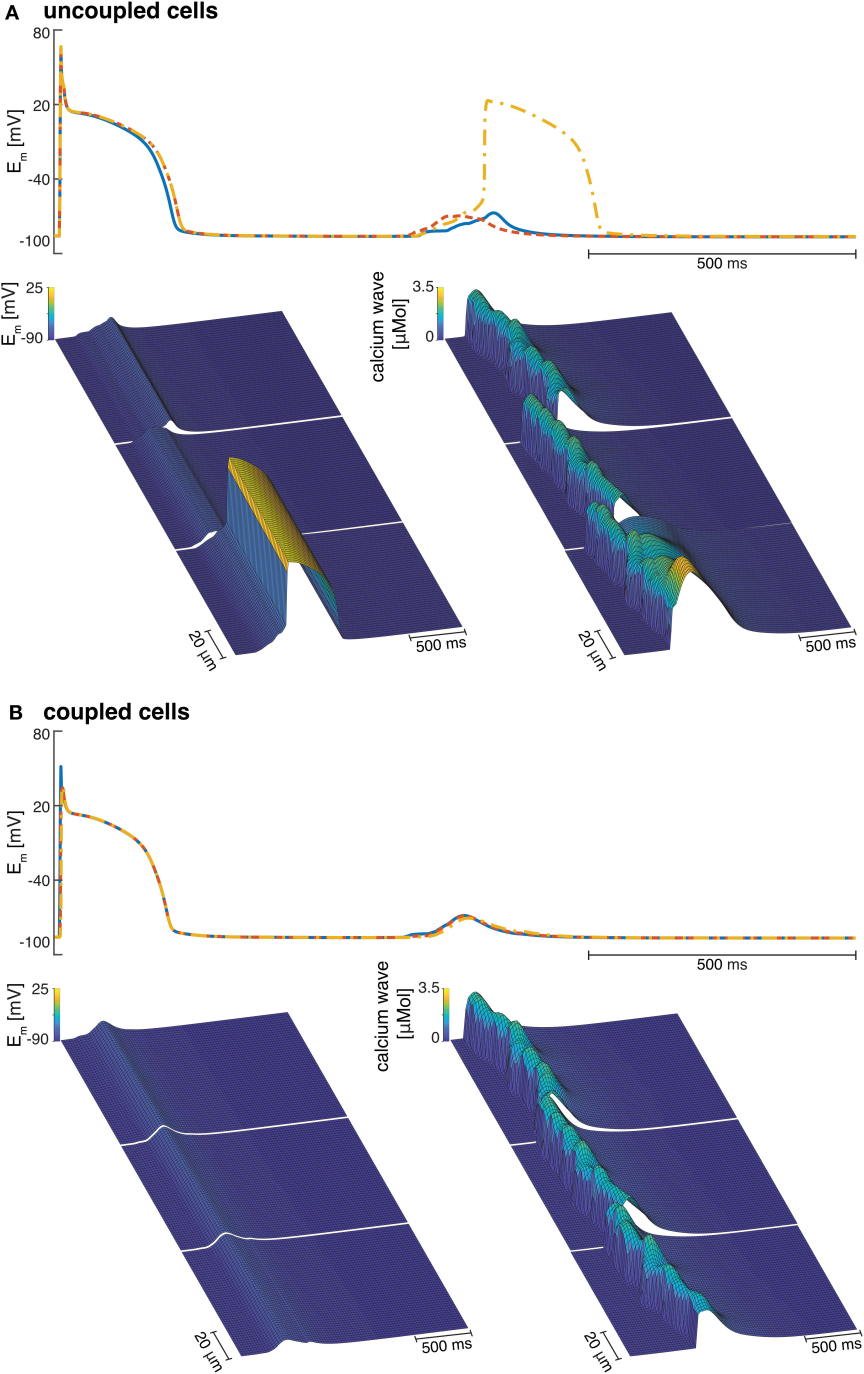
**(A)** Examples of three isolated myocyte APs of the last stimulated beat and the calcium mediated DAD (top), a detailed view of the DAD (bottom left), and the calcium waves in three single cardiomyocytes coupled to three fibroblasts each (bottom right). **(B)** Example of the APs of three coupled myocytes showing the last stimulated beat and the DAD (top), a detailed view of the DAD (bottom left), and the calcium waves in three coupled cardiomyocytes coupled to three fibroblasts each (bottom right).

Electrotonic coupling of cardiomyocytes reduced inter-cellular dyssynchrony of intra-cellular calcium dynamics for all 10 coupled cell replicates. As shown in Figure 7B, we measured more synchronized DAD rise times in multi-cellular compared with isolated cell simulations (see Figure 7A). Current conduction of triggered suprathreshold DADs *via* gap junctions triggered calcium waves with synchronized time-to-peak calcium and wave propagation velocities in all three cells. For coupled cells, the inter-cellular dyssynchrony of intra-cellular calcium dynamics was 0 within the numerical range. By contrast, the dyssynchrony of calcium wave velocity and the DAD rise times of isolated cells varied up to 378.56 ± 170.71 $\frac{\mu \text{m}}{\text{s}}$ and 36.46 ± 23.45 ms, respectively. However, DAD magnitude decreased in coupled cell simulations compared with isolated myocytes due to the greater synchrony.

## 4. Discussion

This computational study examined the importance of MEC, and particular, myofilament-triggered calcium release in the context of chronic stretch. We have assessed the arrhythmogenic potential of four different stretch patterns at cellular and multi-cellular scales with and without coupled fibroblasts. Our results support the hypothesis that MEC might be pro-arrhythmic but only under specific conditions of stretch and calcium overload.

Our model suggests that healthy, isotonically contracting myocytes may be less susceptible to generating propagating calcium waves than isometric contracting myocytes, while stretched myocytes are the most susceptible to calcium waves. For myocytes that do not contract but only stretch over the duration of the twitch (stretch pattern P), the extracellular calcium concentration necessary to trigger propagating waves was 16% lower than in isotonically contracting myocytes. Nevertheless, an increase in extracellular calcium concentration (Ca_o_ ≥ 3.7 mM) was necessary for all stretch patterns to trigger calcium waves. When fibroblasts were coupled to myocytes, electrical charge was drained from the myocytes reducing APD_90_ during normal pacing and slowing calcium waves.

In simulations of myocytes coupled *via* gap junctions, the calcium wave was synchronized between cells but at a lower amplitude compared with those in the single cell simulations. Cell-cell coupling reduced DAD magnitudes, but synchronized the rise times of the triggered suprathreshold DADs due to current conduction *via* gap junctions.

### 4.1. Sub-cellular Structure and Calcium Handling

Spontaneous calcium release events have not only been associated with a calcium overloaded SR (Takamatsu and Wier, 1990; Williams et al., 1992; Cheng et al., 1993, 1996; Satoh et al., 1997; Cheng and Lederer, 2008) but also as the site of calcium wave initiation (Cheng et al., 1996). An increased frequency of calcium sparks is thought to contribute to the initiation of DADs and cellular triggered activity (Voigt et al., 2013). The wavefront of slowly propagating calcium waves are the result of discrete calcium releases from the SR, which recruit other sparks during propagation (Cheng et al., 1996). The incidence of calcium sparks, and thus propagating waves, has also been associated with mechanical heterogeneities (Miura et al., 1993, 1999; Wakayama et al., 2001; ter Keurs et al., 2006a, 2008). Experiments in damaged cardiac muscle discovered that mechanical strain can modify calcium wave velocity resulting in an increase in the propagation velocity (ter Keurs et al., 1998). Consistent with experimental and computational observations, calcium wave velocity affects the amplitude of DADs, which can act as the initiator of arrhythmia at the tissue level (Daniels and ter Keurs, 1990; Daniels et al., 1991; Miura et al., 1993; ter Keurs et al., 2008). Indeed, experiments have shown that premature beats can result from propagating calcium waves that originate close to the border zones of mechanically-heterogeneous tissues (Boyden et al., 2003; Boyden and ter Keurs, 2005; ter Keurs and Boyden, 2007). Therefore, MEC may be an important contributor to ventricular arrhythmia in cardiac pathologies associated with mechanical perturbations.

The patterns of systolic stretch used in our simulations are similar to those described in regions of myocardial mechanical heterogeneity. Experimental studies of acute ischemia (Baumeister et al., 2018) revealed that ischemic tissue shows a similar stretches as stretch pattern P used in for this computational study. Late diastolic stretches, such as stretch pattern S, have been observed in various pathological conditions such as ischemia or heart failure (Neves et al., 2016) and isometric conditions are often used in experimental set-ups to immobilize the cells for optical mapping. In pathological conditions, such as ischemia, stretch is assumed to enhance calcium spark probability (Cameron et al., 2020), which may trigger calcium waves and DADs originating from these sites (Boyden et al., 2003; Boyden and ter Keurs, 2005; ter Keurs and Boyden, 2007). In addition to calcium waves, spontaneous contractile waves have been related to pro-arrhythmic effects in arrangements of weaker damaged regions in-series with normal muscle segments. Even though the underlying arrhythmogenic mechanism is thought to be caused by calcium release, the initiation of the calcium release has been linked to stretch and release of the myofilaments. The increase in intra-cellular calcium may be conducted to adjacent myocytes triggered by tissue-level heterogeneities in mechanical loading the resulting spontaneous contractile waves (ter Keurs et al., 1998).

### 4.2. Comparison With Previous Models

Cardiomyocyte function is tightly regulated by calcium signaling mechanisms that are well studied. While fibroblasts are also regulated by calcium fluxes (Feng et al., 2019), the mechanisms of calcium homeostasis in these non-excitable cells, and the role of voltage-gated calcium channels are less well studied and modeled (Feng et al., 2019). Mathematical models have not yet incorporated calcium fluxes between coupled myocytes and fibroblasts (MacCannell et al., 2007; Maleckar et al., 2009), and detailed models of calcium handling in fibroblasts are independent of membrane potential (Kotwani et al., 2014; Kotwani, 2015). Thus, there is a need for better models of fibroblast calcium fluxes and their regulation by transmembrane potential.

Spontaneous calcium release has been studied extensively in experiments (Capogrossi and Lakatta, 1985; Sasse et al., 2007; Rapila et al., 2008) as well as computational studies (Means et al., 2006; Dupont et al., 2007; Solovey et al., 2008). To study myocyte calcium handling, several computational models have been developed ranging from 0D to three-dimensional (3D) implementations and from highly detailed molecular models to common pool models (Means et al., 2006; Dupont et al., 2007; Solovey et al., 2008). Since calcium handling abnormalities are often related to cardiac diseases as heart failure, models have also focused on ryanodine receptor (RyR) and L-type channel dysfunction. Both, dysfunction or location heterogeneities of RyR clusters and L-Type channels have been associated with an impact on spontaneous calcium release events, and thus, impairment of calcium waves or pro-arrhythmic calcium waves (Zahradńıková and Zahradńık, 2012; Walker et al., 2014). However, to our knowledge none of these models include MEC to study the effects of mechanical heterogeneities in cells or tissues.

In larger tissue scale studies gap junctions play an important role. While our study only considered a linear gap junction model, various models of more detailed static and dynamic gap junction models have previously been developed. Vogel and Weingart (1998) developed a static model of gap junctions in which the voltage- and time-dependent conductance changes based on the voltage in myocyte pairs. A more recent dynamic model by Henriquez et al. (2001) describes each gap junction as two hemichannels in-series, which exist in either a high or low conductance state. Hence, the model assumes that the conductive gap junction channels have four conformational states regulated by nonlinear functions of the junctional voltage (Oka et al., 2006). Experimental data (Oka et al., 2006) also suggest that gap junction gating is regulated by intra-cellular calcium and protons. While a more detailed gap junction model would produce more physiological results than our linear model, they are also more computationally expensive.

### 4.3. Limitations

Our model was unable to elicit calcium waves at extracellular calcium concentrations below 3.7 mM, similar to some experimental studies that have used unphysiologically high extracellular calcium concentrations (Capogrossi et al., 1986a; Cheng et al., 1996). However, this is a limitation of the model because other experimental studies have observed calcium waves at concentrations as low as 1.8–2.0 mM (Takamatsu and Wier, 1990; Wier and Blatter, 1991). There are several aspects of the calcium handling mechanisms in our model that might account for the limitation that high extracellular calcium concentrations were needed to elicit propagating calcium waves. In particular, the present model does not incorporate calcium fluxes between myocytes and fibroblasts, which have been reported in experiments (Feng et al., 2019) and have been modeled mathematically by one group (Kotwani et al., 2014; Kotwani, 2015).

Our model suggests that specific chronic stretch patterns are likely to have a pro-arrhythmic effect under calcium overload conditions. However, the myocyte model only considered a 1D representation in longitudinal direction. Therefore, the model cannot give insights into cellular anisotropy and radial wave propagation (Galice et al., 2018). Additionally, we only consider sarcomeric heterogeneity but neglect possibly important details about sub-cellular distribution of RyR clusters and L-type channels, which might have a strong impact on the calcium handling (Sutanto et al., 2018). Furthermore, our conclusions are based on a deterministic model while stochastic approaches are necessary to reproduce heterogeneous systems as RyR clusters which can open spontaneously. Therefore, our model could not generate propagating waves that do not activate the whole myocyte.

The myofilament contraction model (Rice et al., 2008b) was chosen in part because of its computational efficiency compared with spatial explicit sarcomere models. It was built largely with steady-state data and a simplified representation of calcium binding to the myofilaments, while more recent models allow for a more realistic representation of this process. The parameterization of calcium diffusion in the EP model (Shannon et al., 2004) was based on ECC data and can only partially reproduce MEC.

Finally, in this study we only considered one MEC mechanism, even though mechano-sensitive ion channels and XROS are also known to contribute to mechanics-induced arrhythmia (Kohl et al., 2011). Nevertheless, mechanically-induced calcium waves have also been reported when mechano-sensitive ion channels were blocked (Wakayama et al., 2001). XROS on the other hand might be important in some context, especially during diastolic stretch (Prosser et al., 2011, 2013). But we did not explicitly simulate XROS since we used whole muscle data for the parameterization of the myofilament-triggered calcium release events.

## 5. Conclusions

In this study, we investigated the ability of sub-cellular mechanical transients and heterogeneity and the effects of coupled fibroblasts to modify calcium wave velocity and the resulting calcium mediated DADs. The goal was to investigate whether MEC, transient stretches, and fibroblasts contribute to pro-arrhythmic effects in calcium overloaded myocytes and multi-cellular tissues.

The present study indicates that myofilament-triggered calcium release mechanisms may modulate the susceptibility threshold for DADs. Chronic stretch may increase cytosolic calcium concentration resulting in an increase of the magnitude of the calcium-mediated DAD. In contrast, myocyte coupled to fibroblasts are less susceptible to calcium-mediated DADs as the current through the gap junction between the myocyte and the fibroblasts may provide a drain of the electric charge. Finally, at the multi-cellular scale, mechanically-induced calcium waves may trigger synchronized calcium waves and after-depolarizations. Thus, MEC may increase the susceptibility to pro-arrhythmic intra- and inter-cellular calcium wave propagation.

## Data Availability Statement

All datasets generated for this study are included in the article/Supplementary Material.

## Author Contributions

VT carried out all simulations, analyzed the data, and wrote the article. Together with VT, AM designed the research. AM commented and edited the manuscript. All authors contributed to the article and approved the submitted version.

## Conflict of Interest

AM is a co-founder of and has an equity interest in Insilicomed and Vektor Medical. He serves on the scientific advisory board of Insilicomed and as scientific advisor to both companies. Some of his research grants, including those acknowledged here, have been identified for conflict of interest management based on the overall scope of the project and its potential benefit to these companies. The remaining author declares that the research was conducted in the absence of any commercial or financial relationships that could be construed as a potential conflict of interest.
