# Supplementary material for: Mechano-Electric Coupling and Arrhythmogenic Current Generation in a Computational Model of Coupled Myocytes
Source: Front Physiol. 2020 Dec 10;11:519951. doi: 10.3389/fphys.2020.519951 (PMC7758443; doi:10.3389/fphys.2020.519951)
Supplement: Supplementary file 1 [file Data_Sheet_1.pdf]

SUPPLEMENTAL DATA

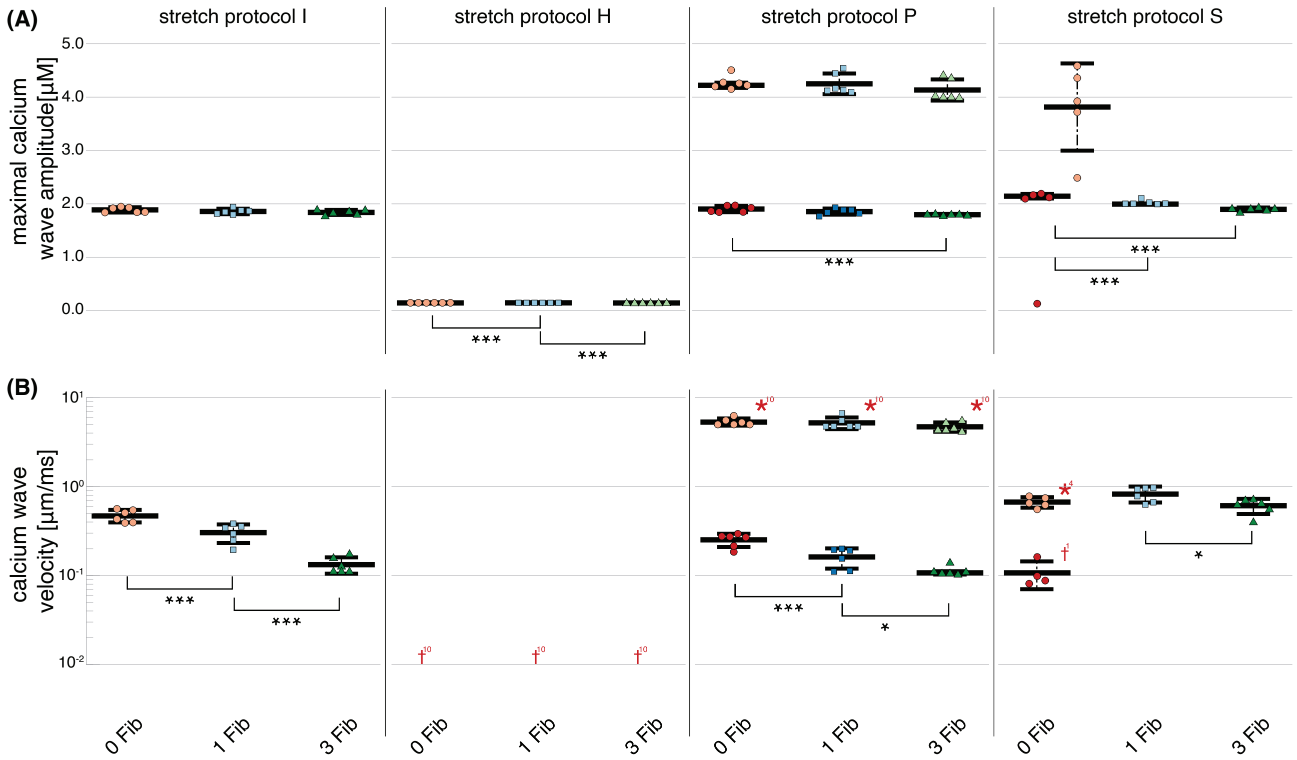

Figure 1. Maximal calcium wave amplitude and calcium wave velocity for simulations of 6 coupled cardiomyocytes. (A) For all stretch patterns but H (from left to right: control, S, and P) the maximal calcium wave amplitude significantly ($p<0.01$) decreased the more fibrobasts were coupled to the multi-cellular string (control red data points, 1 coupled fibroblast blue data points, and 3 coupled fibroblasts green data points; the data points in light colors specify the first spontaneous event and dark colors are used for the following spontaneous event.) (B) The wave velocity significantly ($p<0.01$) decreased for all stretch patterns from no coupled fibroblast to 3 coupled fibroblasts.

	CRU	1	2	3	4	5	6	7	8	9	10
sarcomere length [μm]	1	1.8587	1.9446	2.0650	1.7235	1.8149	1.8609	2.0472	1.9211	1.9793	2.0226
	2	1.7198	1.8441	1.9077	1.9588	1.9660	1.7366	1.7014	1.9054	1.8502	1.9129
	3	2.0422	2.0078	1.8980	1.7170	1.9529	1.9272	1.8758	2.0298	1.9593	2.0524
	4	2.0581	1.9024	1.7885	1.7280	1.9048	1.8790	1.8614	1.8011	1.9671	1.9643
	5	1.8865	1.8336	1.8858	1.8982	1.9649	1.9641	1.8752	1.8212	1.8682	1.9213
	6	1.8859	2.0559	1.9369	1.7376	1.9529	1.9656	1.9921	1.7461	1.7084	2.0092
	7	1.8287	2.0321	1.9577	2.0103	1.7683	1.9424	1.8229	2.0563	1.8261	2.0333
	8	2.0412	1.9090	1.8505	2.0100	1.7494	1.7137	1.9976	1.9450	1.8614	2.0748
	9	1.8406	1.9363	1.8399	1.9741	2.0787	1.7270	1.8792	1.8822	1.8032	1.7012
	10	1.7430	1.9229	2.0745	1.7576	1.7657	1.8218	1.7145	1.9427	1.7755	2.0281
	11	1.9959	1.7795	1.7153	1.9503	1.7133	1.9017	1.7675	1.9069	2.0116	1.9326
	12	1.8483	1.8149	2.0356	1.8970	1.9131	1.9484	1.9738	1.9457	1.8635	2.0752
	13	1.7924	1.8790	2.0462	2.0688	2.0343	1.8551	1.8800	1.9066	2.0366	1.9005
	14	1.8537	1.7881	2.0020	1.9463	1.9539	2.0110	1.7587	1.9736	1.8489	1.8823
	15	1.7375	2.0201	1.7383	2.0035	1.7730	1.9725	1.8299	1.8985	1.9917	2.0039
	16	1.7509	1.7746	1.8000	1.8725	1.8405	2.0671	1.9306	2.0766	1.8510	1.7871
	17	2.0571	1.7864	1.8278	1.8644	1.8752	1.9018	1.7735	1.7837	2.0066	1.8893
	18	2.0624	1.7655	1.9579	2.0130	2.0721	1.8239	1.9801	1.7410	1.9864	2.0415
	19	1.9184	1.7871	1.7526	1.7326	1.7601	1.7409	1.7928	1.7425	1.8437	1.9182
	20	1.7236	1.8657	1.9736	1.7513	2.0244	1.9319	2.0478	1.7250	1.7827	2.0205
	21	1.7897	1.8186	1.7414	1.7665	1.9447	1.9954	1.8027	1.8539	1.9998	1.9802
	22	1.8345	2.0500	1.9481	1.8488	1.8432	1.8611	1.9904	1.8705	2.0598	1.9225
	23	2.0114	1.8636	1.8878	2.0153	1.7732	1.7353	1.7723	1.8393	1.8248	1.7943
	24	1.7068	1.7709	1.9955	2.0047	1.8629	1.8017	1.8097	1.9896	1.9547	1.9529
	25	1.7173	2.0430	1.9713	1.7239	1.8832	1.7591	1.7354	1.9383	1.8668	1.7326
	26	1.7649	2.0713	2.0426	1.8519	1.7466	1.8072	1.9188	1.9928	2.0161	1.9376
	27	1.9464	1.8669	2.0378	1.9002	1.9238	1.8674	1.9593	2.0536	1.9916	1.9508
	28	1.9776	1.7430	1.8273	1.8586	1.7865	1.9003	1.9076	2.0687	1.7642	1.9768
	29	1.9458	1.7985	1.9651	1.9493	1.8464	1.8739	1.8619	1.7736	2.0268	2.0377
	30	1.8714	1.8555	1.7758	1.9384	1.9214	2.0319	1.9446	1.7535	2.0752	2.0723
	31	1.9078	1.9259	1.7125	1.8114	1.7962	1.8968	1.9458	1.9642	1.8955	1.9917
	32	1.8130	1.8001	1.9823	1.8642	1.8108	2.0577	1.9577	1.7365	2.0353	1.9208
	33	1.9825	1.9289	1.8900	1.7069	1.9343	1.9421	1.9413	1.8996	1.9233	2.0519
	34	1.7724	1.9698	1.8824	2.0730	1.8013	2.0630	2.0583	1.9015	1.7595	1.9203
	35	1.9606	1.7848	2.0430	1.7642	2.0126	1.7920	1.7800	2.0265	1.7765	1.7074
	36	1.7704	1.7454	1.9315	1.7411	2.0724	1.9566	1.9691	1.8843	1.8548	1.7467
	37	1.8403	1.8131	1.9345	1.8418	1.9770	1.8103	1.7903	1.8497	1.9840	2.0271
	38	1.9375	1.8215	2.0259	1.7759	1.8310	1.9549	1.7461	1.9548	2.0131	1.8841
	39	1.9959	1.8613	2.0055	1.8861	1.9218	1.9638	1.9306	1.9812	1.9996	2.0204
	40	1.7317	1.8930	1.9190	1.8293	1.7417	1.7267	1.8712	1.8976	1.8214	1.7802
	41	2.0523	1.7333	1.7701	2.0607	2.0436	1.7973	1.8744	1.8324	1.9029	1.9098
	42	1.9942	1.8002	1.7917	2.0489	2.0335	1.7857	1.9512	1.7577	1.7350	1.9391
	43	1.8850	2.0038	2.0361	1.7209	2.0101	1.9534	1.9922	1.9225	1.7432	1.7131
	44	1.8658	1.7120	1.7118	1.9799	1.7996	2.0202	1.8334	1.8001	1.7525	1.9334
	45	1.8699	2.0521	1.8862	1.8027	1.9257	1.8312	1.9512	1.7178	1.9575	1.8380
	46	1.8168	1.9771	1.7645	1.8608	1.7095	1.9960	1.8583	1.9864	1.8882	1.7197
	47	1.8932	1.8857	2.0709	1.9081	1.8617	1.9563	2.0192	1.7928	1.7727	1.8861
	48	1.8941	1.9197	1.9704	2.0574	1.8192	1.7035	2.0158	1.8682	1.8881	1.7738
	49	2.0101	1.7907	1.8902	1.8589	1.7620	1.9286	1.7979	1.9610	1.7568	1.7475
	50	2.0014	1.8744	1.8791	2.0726	1.7686	1.8472	1.9329	1.8368	1.7218	1.7787

Table 1. Sarcomere length distribution for the the different cardiomyocyte simulations.

	myocyte	CRUs	fibroblast conduction [SI]
Fib 1	1	1	4.6041
	2	49	2.4313
	3	5	6.6071
	4	2	3.1249
	5	2	5.1203
	6	48	6.7312
	7	4	7.379
	8	3	6.153
	9	3	1.0689
	10	50	6.3438
Fib 3	1	46, 47, 50	1.2999, 3.8201, 1.0863
	2	1, 2, 46	2.2994, 1.0698, 1.4249
	3	2, 4, 49	3.0329, 4.1694, 4.1815
	4	2, 46, 50	1.4898, 3.5293, 1.2234
	5	3, 4, 47	0.82268, 6.659, 0.61553
	6	4, 47, 50	6.0852, 1.9172, 1.9172
	7	3, 46, 47	4.1509, 3.7689, 3.8509
	8	2, 5, 48	4.4962, 3.3396, 4.4962
	9	1, 3, 49	1.8861, 7.2866, 7.8481
	10	2, 3, 50	5.0213, 5.8341, 2.1631

Table 2. Calcium-release unit (CRU) location and conduction of coupled fibroblast for each of the 10 different myocytes.

	Ca_o [mM]	stretch protocol I	stretch protocol P	stretch protocol H	stretch protocol S
max calcium wave amplitude [µM]	2.0	0.1005 ± 0.0002	0.1087 ± 0.0006	0.0968 ± 0.0003	0.1003 ± 0.0003
	first occurring wave	1.2460 ± 0.8190	0.7030 ± 0.8141	1.5848 ± 0.5392	1.4382 ± 0.7263
	4.2	1.8963 ± 0.0520	4.4799 ± 0.1216<br>1.9396 ± 0.0620	0.1469 ± 0.0009	3.1256 ± 0.6926<br>0.5283 ± 0.7225
calcium wave velocity [µm/s]	2.0	no waves	no waves	no waves	no waves
	first occurring wave	104.5 ± 03.9	100.2 ± 7.0	101.6 ± 004.5	106.3 ± 006.2
	4.2	318.5 ± 057.9	4601.7 ± 432.6<br>175.6 ± 38.0	no wave	786.7 ± 85.5<br>115.9 ± 19.8

Table 3. Mean and standard deviation for the maximal calcium wave amplitude magnitude and calcium wave velocity in cardiomyocyte simulations at different extracellular calcium concentrations for control (stretch protocol H), isometric contraction (stretch protocol I), and stretch patterns S and P.

	fibroblasts	stretch protocol H	stretch protocol I	stretch protocol P	stretch protocol S
APD_{90} [ms]	0	216.0 ± 0.0005	158.0 ± 0.0005	213.0 ± 0.0005	115.0 ± 0.0005
	1	163.8 ± 0.4412	199.0 ± 0.0004	215.0 ± 0.0	217.1 ± 0.3333
	3	221.6 ± 5.2706	214.9 ± 0.3332	162.8 ± 2.6353	228.1 ± 5.7974

Table 4. Mean and standard deviation of the APD_{90} in cardiomyocyte simulations with 0, 1, and 3 coupled fibroblasts for control (stretch protocol H), isometric contraction (stretch protocol I), and stretch patterns S and P.

	fibroblasts	stretch protocol H	stretch protocol I	stretch protocol P	stretch protocol S
max calcium wave amplitude [µM]	0	0.1468 ± 0.0008	1.9016 ± 0.0518	4.4623 ± 0.1275 1.9396 ± 0.0620	3.1256 ± 0.6926 0.4960 ± 0.6888
	1	0.1455 ± 0.0008	1.8517 ± 0.0312	4.4030 ± 0.1288 1.8550 ± 0.0856	2.3273 ± 0.5194
	3	0.1455 ± 0.0072	1.6315 ± 0.5202	4.2873 ± 0.1347 1.2179 ± 0.7494	1.9636 ± 0.0616
calcium wave velocity [µm/s]	0	no wave	326.8 ± 60.6	4596.1 ± 408.2 175.6 ± 38.0	786.7 ± 85.5 23.2 ± 49.3
	1	no wave	176.8 ± 36.9	4359.4 ± 397.9 141.9 ± 31.7	747.9 ± 120.6
	3	no wave	111.0 ± 5.8	4005.5 ± 408.8 111.8 ± 16.8	494.9 ± 086.7

Table 5. Mean and standard deviation for the maximal calcium wave amplitude magnitude and calcium wave velocity in cardiomyocytes coupled to 0, 1, 2, and 3 fibroblasts evaluated for control (stretch protocol H), isometric contraction (stretch protocol I), and stretch patterns S and P.

	1	2	3	4	5	6	7	8	9	10
3 coupled myocytes	1, 2, 3	10, 5, 6	4, 7, 9	6, 3, 7	7, 1, 4	2, 10, 8	, 5, 6, 1	8, 9, 2	7, 4, 3	3, 7, 5

Table 6. Number and order of the coupling of the single cardiomyocytes from Tables 1 and 2 for the simulation of 6 coupled myocytes *via* gap junctions.

	1	2	3	4	5
6 coupled myocytes	8, 7, 1, 4, 9, 10	6, 3, 4, 1, 9, 2	3, 7, 8, 9, 1, 10	5, 3, 6, 8, 2, 1	2, 9, 8, 4, 6, 3

Table 7. Number and order of the coupling of the single cardiomyocytes from Tables 1 and 2 for the simulation of 6 coupled myocytes *via* gap junctions.
